# Naringin Supplementation during Pregnancy Induces Sex and Region-Specific Alterations in the Offspring’s Brain Redox Status

**DOI:** 10.3390/ijerph18094805

**Published:** 2021-04-30

**Authors:** Bernardo Gindri dos Santos, Caroline Peres Klein, Mariana Scortegagna Crestani, Rafael Moura Maurmann, Régis Mateus Hözer, Karoline dos Santos Rodrigues, Pauline Maciel August, Cristiane Matté

**Affiliations:** 1 Programa de Pós-Graduação em Ciências Biológicas: Bioquímica, Instituto de Ciências Básicas da Saúde, Universidade Federal do Rio Grande do Sul, Porto Alegre 90035-000, Brazil; carolpkl@gmail.com (C.P.K.); regishozer@gmail.com (R.M.H.); rodrigues206@outlook.com (K.d.S.R.); maciel.pauline@gmail.com (P.M.A.); matte@ufrgs.br (C.M.); 2 Departamento de Bioquímica, Instituto de Ciências Básicas da Saúde, Universidade Federal do Rio Grande do Sul, Porto Alegre 90035-000, Brazil; marianascrestani@gmail.com (M.S.C.); rafael.maurmann@acad.pucrs.br (R.M.M.); 3 Programa de Pós-Graduação em Ciências Biológicas: Fisiologia, Instituto de Ciências Básicas da Saúde, Universidade Federal do Rio Grande do Sul, Porto Alegre 90050-170, Brazil

**Keywords:** flavonoids, pregnancy, DOHaD, brain, redox status, antioxidant

## Abstract

Research has shown the beneficial effects of naringin supplementation to adult rodents, which can ameliorate oxidative stress in disease models. However, evidence has demonstrated that polyphenol supplementation induced detrimental effects when consumed during sensitive periods of development, such as pregnancy. Therefore, we investigated the effect of maternal naringin supplementation during pregnancy on the offspring’s cerebral redox status. Pregnant Wistar rats were divided into control and naringin groups and supplemented from gestational day 15 to gestational day 21. On postnatal days 1, 7, and 21, offspring were euthanized, and the prefrontal cortex, hippocampus, striatum, and cerebellum dissected. On postnatal day 1, maternal naringin supplementation positively modulated the pups’ brain redox status. On postnatal day 7, a pro-oxidative milieu was observed in the offspring’s striatum and cerebellum in a sex-dependent manner, even though the prefrontal cortex and hippocampus were not negatively affected. Besides, the alterations observed on postnatal day 7 did not persist up to weaning. Our findings demonstrated that the effect induced by naringin supplementation in the brain redox status differed according to the period of development in which naringin was consumed since the beneficial effects usually found in the adult rodents became detrimental when the supplementation was applied during pregnancy.

## 1. Introduction

Naringin, which is a glycosylated flavonoid, is commonly found in Chinese herbal medicines, citrus fruits, and derivative beverages [1]. During the past years, it has been demonstrated that naringin can promote neuroprotection in experimental models of cognitive dysfunction, such as Parkinson’s disease, epilepsy, and Alzheimer’s disease mainly by reducing oxidative stress and improving neuronal survival [2,3,4,5]. Additionally, though data from clinical and observational research is still limited, they also show that citrus fruit extract, grapefruit, and even naringin supplements can reduce total cholesterol, low-density lipoprotein (LDL), and improve blood pressure [6,7,8,9]. Therefore, this whole body of evidence suggests that flavonoid intake, including naringin, can induce beneficial effects and promote health in the general population when consumed in the diet or by flavonoid-based supplements.

Accordingly, herbal supplements, which contain flavonoids in their composition, are commonly used and perceived as safe by the general population mainly because they can be purchased without medical prescription and are derived from natural products, such as fruits, teas, or herbs [10,11,12,13]. Other reports have also demonstrated that the consumption of polyphenol-rich supplements in specific population groups, such as pregnant women, ranges from 28.9% to 57.8% in different countries, mostly stimulated by friends, family, or their own initiative [14,15,16,17]. Taken together, this data suggests a misconception by the general population regarding polyphenol-rich supplements safety, which is a situation particularly delicate because of two reasons: the polyphenol doses reached by supplementation are many times higher than what is normally found in the diet, and the second is the lack of data from studies evaluating the safety of polyphenol supplementation during pregnancy for the mother and the developing fetus [18,19].

In this context, the Developmental Origins of Health and Disease (DOHaD) field of study aims to understand how interventions during critical periods of development, such as pregnancy, might induce alterations in the progeny’s development [20]. In recent years, an increasing body of evidence from animal studies has been demonstrating that maternal diet interventions during pregnancy, such as polyphenol supplementation, can induce postnatal alterations in the offspring’s blood biochemical parameters, antioxidant status, inflammatory status, and cholesterol transport in different tissues [21,22,23,24,25]. Additionally, epigallocatechin gallate, a flavonoid commonly found in green tea, was able to inhibit migration and adhesion of neural progenitor cells in vitro, thereby suggesting that polyphenols might also interfere during neurodevelopment [26].

Moreover, the impact of maternal high polyphenol intake was also reported in human studies, though they are still very scarce. ZIELINSKY et al. (2010) found an association between high maternal intake of polyphenol-rich foods during late pregnancy with alterations in fetal *ductus arteriosus* flow dynamics, which can potentially impair cardiovascular health during postnatal development [27]. The authors also demonstrated that the inflammatory milieu and the increased nitric oxide content, both necessary for the ductus development during late pregnancy, were counteracted by the high maternal polyphenol intake, which altered the correct development and closure of the *ductus arteriosus* [28,29]. Besides demonstrating that the maternal high polyphenol intake during pregnancy could modify the redox status in the fetal heart, the authors also showed that the effects induced by the high polyphenol consumption in the ductus closure dynamics were observed only if the consumption occurred during the third week of pregnancy. These data suggest that dietary interventions during such periods of development can induce long-lasting effects during the progeny’s postnatal life.

Therefore, considering the evidence suggesting that: (1) maternal high polyphenol intake can modify the redox status in fetal tissues, (2) naringin is a redox-active molecule able to modulate brain redox status, and (3) interventions during an organ’s critical window of development can induce persistent alterations, we hypothesized that maternal naringin supplementation during the third week of pregnancy can modify the offspring’s brain redox homeostasis during postnatal development. Here, the prefrontal cortex, hippocampus, striatum, and cerebellum were evaluated at three different postnatal ages up to postnatal day 21 in both sexes. By these means, we also aimed to assess if the alterations induced by prenatal naringin exposure could be sex-dependent and if they would persist along with postnatal development.

## 2. Materials and Methods

### 2.1. Ethical Standards

The experimental design and procedures were approved by the local Ethics Commission on Animal Use (CEUA/UFRGS), under protocol number 35332. All experiments follow the ARRIVE guidelines and were performed in accordance with the National Animal Rights Regulations (Law 11.794/2008), the American National Institute of Health Guide for the Care and Use of Laboratory Animals (NIH publication No. 80-23, revised 1996), and the Directive 2010/63/EU.

### 2.2. Animals

Pregnant Wistar rats (90 days-old) were obtained from the animal facility at Departamento de Bioquímica, Instituto de Ciências Básicas da Saúde, Universidade Federal do Rio Grande do Sul (UFRGS), Porto Alegre, RS, Brazil. The animal facility was under controlled light (12:12 h light/dark cycle), temperature (22 ± 1 °C), and humidity conditions (50–60%). Animals had free access to a 20% (*w*/*w*) protein commercial chow and water ad libitum.

### 2.3. Reagents

Purified naringin was obtained from Sigma-Aldrich Chemical Co. (St. Louis, MO, USA). We chose to use purified naringin to evaluate its isolated effect. This flavonoid is commonly found in citrus fruits that are widely consumed in the human diet, such as lemon, oranges, grapefruit, and its derivatives [30,31]. Moreover, purified naringin supplements are currently available online for purchase.

### 2.4. Experimental Protocol

The female Wistar rats were mated in a proportion of 1 male to 2 females per cage, and the pregnancy diagnosis was confirmed by the presence of sperm in the vaginal smear and it was considered as the gestational day zero [32]. Pregnant rats were randomly divided into two experimental groups treated by oral gavage: control (equivalent volume of distilled water) and naringin groups (100 mg/kg/day). This dose was selected based on studies that have demonstrated modulation of the redox status by naringin in different brain regions in adult rodents [33,34].

The naringin supplementation occurred during the third week of pregnancy, between gestational days 15 and 21. Such period was chosen based on reports demonstrating that the intrauterine development of the brain regions analyzed here predominantly occurs during the third week of gestation; therefore, the maternal naringin supplementation was administered during such a critical window of development [35,36,37]. The delivery date was considered as postnatal day 0. After delivery, the pups were randomly selected and culled to maintain litters of eight pups per dam with the same proportion of males and females. From delivery up to weaning each dam was housed with its own litter.

On postnatal days 1, 7, and 21 the male and female pups were randomly picked from each control and naringin litter and euthanized by decapitation without anesthesia to avoid tissue chemical contamination. The prefrontal cortex, hippocampus, striatum, and cerebellum were dissected and stored at −80 °C. Here we considered the litter as the experimental unit; therefore, all the analyses were undertaken with samples from pups obtained from different litters to avoid the litter effect [38]. For each biochemical analysis, we have used between 6 and 8 pups as the experimental “*n*”.

### 2.5. Sample Processing

The brain regions were homogenized (1:10 *w*/*v*) in 20 mM sodium phosphate buffer (Sigma Chemical Co., St. Louis, MO, USA), pH 7.4, containing 140 mM KCl (Sigma Chemical Co., St. Louis, MO, USA), and centrifuged at 750× *g* for 10 min at 4 °C. The pellet was discarded, and the supernatant was used for the biochemical analyses.

### 2.6. Biochemical Analyses

#### 2.6.1. Total Oxidants Level

2′,7′-dichlorofluorescein (DCFH) () oxidation was evaluated fluorometrically in a 96-well plate. Briefly, 50 µL of the diluted sample homogenate was incubated with 200 µL H_2_DCF-DA (Sigma Chemical Co., St. Louis, MO, USA) at 37 °C for 30 min in a dark room. DCFH converted from H_2_DCF-DA, was oxidized further by cellular oxidants, mainly H_2_O_2_, producing DCF, which is a fluorescent compound. DCF fluorescence was detected at 488 nm excitation and 525 nm emission. A DCF standard curve from 0.25 to 10 mM was also used in parallel. Data are expressed as nmol/mg protein [39].

#### 2.6.2. Antioxidant Enzymes Assays

The antioxidant enzyme activities were evaluated as described below. Moreover, the SOD/GPx ratio was mathematically calculated by dividing the SOD activity by the GPx activity of the same sample in the following formula: SOD/GPx ratio = (SOD activity/GPx activity) × 100 [40].

#### 2.6.3. Superoxide Dismutase Activity

Superoxide dismutase (SOD, EC 1.15.1.1) activity was evaluated by quantifying the inhibition of the autoxidation of epinephrine by SOD at 480 nm. The reaction medium consisted of 50 mM glycine buffer (Sigma Chemical Co., St. Louis, MO, USA) pH 10.2 containing 0.01 mM of bovine catalase (Sigma Chemical Co., St. Louis, MO, USA), and 0.05 mM of epinephrine (Sigma Chemical Co., St. Louis, MO, USA) as the reaction starter. Total SOD activity is expressed as the amount of enzyme that inhibits the oxidation of epinephrine by 50%, which is equal to one unit. The data were calculated as units/mg protein [41].

#### 2.6.4. Glutathione Peroxidase Activity

Glutathione peroxidase (GPx, EC 1.11.1.9) activity was evaluated by the decrease of NADPH (Sigma Chemical Co., St. Louis, MO, USA) concentration at 340 nm. The reaction medium contained 100 mM potassium phosphate buffer (Sigma Chemical Co., St. Louis, MO, USA), pH 7.7, 1 mM EDTA (Sigma Chemical Co., St. Louis, MO, USA), 2 mM reduced glutathione (Sigma Chemical Co., St. Louis, MO, USA), 0.15 U/mL glutathione reductase (Sigma Chemical Co., St. Louis, MO, USA), 0.4 mM azide (Sigma Chemical Co., St. Louis, MO, USA), 0.1 mM NADPH, and 0.5 mM tert-butyl hydroperoxide (Sigma Chemical Co., St. Louis, MO, USA) as enzyme substrate. GPx unit is defined as 1 µmol of NADPH consumed per minute and the specific activity as units/mg protein [42].

#### 2.6.5. Catalase Activity

Catalase (CAT, EC 1.11.1.6) activity was evaluated by measuring the reduction of hydrogen peroxide at 240 nm in a reaction medium containing 20 mM H_2_O_2_ (Sigma Chemical Co., St. Louis, MO, USA), 0.1% Triton X-100 (Sigma Chemical Co., St. Louis, MO, USA), and 10 mM potassium phosphate buffer, pH 7.0. One CAT unit is defined as 1 µmol of H_2_O_2_ consumed per minute and the specific activity as units/mg protein [43].

#### 2.6.6. Glyoxalase 1 Activity

Glyoxalase 1 (GLO1, EC 4.4.1.5) activity was measured by following the increase in the S-D-lactoylglutathione at 240 nm. The reaction medium consisted of 60 mM sodium phosphate buffer, pH 6.6, with reduced glutathione 0.01 M, and methylglyoxal 0.01 M (Sigma Chemical Co., St. Louis, MO, USA). A GLO1 unit is defined as the amount of enzyme needed to catalyze the formation of 1 µmol of S-D-lactoylglutathione per minute, and the specific activity is represented as units/mg protein [44].

#### 2.6.7. Total Reduced Glutathione Content

First, high molecular weight proteins in the tissue homogenate were precipitated with meta-phosphoric acid (1:1, *v*/*v,* Sigma Chemical Co., St. Louis, MO, USA), and centrifuged at 5000× *g* for 10 min at 25 °C. Reduced glutathione (GSH) present in the supernatant reacted with the fluorophore o-phtaldialdehyde (7.5 mM) prepared in 100 mM sodium phosphate buffer, pH 8.0, with 5 mM EDTA. The fluorescence was read at excitation and emission wavelengths of 350 nm and 420 nm, respectively, using the SpectraMax Gemini XS Fluorescence microplate reader (Molecular Devices, Sunnyvale, CA, USA). A standard GSH curve ranging from 0.001 to 1 mM was prepared and a blank sample was performed in parallel. Data were expressed as nmol of GSH/mg protein [45].

#### 2.6.8. Protein Determination

The total protein content of the samples was measured according to LOWRY et al. (1951) modified by PETERSON et al. (1977) and adapted to 96 well plates using bovine serum albumin as the standard [46,47]. Briefly, the samples were diluted in ultra-pure water in a proportion of 1/20 and then incubated with Lowry solution for 10 min under constant agitation of 30 g and temperature of 25 °C. Following the 10 min incubation, the 0.08 M folin solution (Dinâmica, Indaiatuba, São Paulo, Brazil). was added and the samples incubated for 30 min under constant agitation of 30 g and temperature of 25 °C in a dark room. The samples were read at 750 nm wavelength and the protein content was expressed as mg/mL.

### 2.7. Statistical Analysis

The analysis was performed by the IBM SPSS 22.0 program (SPSS Inc., Chicago, IL, USA). The data were tested for normality and the outliers exceeding ±2 standard deviations were excluded when necessary. All data were analyzed by the two-way analysis of variance (ANOVA) with maternal naringin supplementation (*) and the offspring’s sex (#) as the two main independent factors. The ANOVA tests were followed by Sidak’s multiple comparison test when interactions were statistically significant. All data are expressed as mean ± standard error of the mean (S.E.M.) and considered statistically significant when *p* < 0.05. All the descriptive statistical results are available within the Supplementary Materials in Tables S1–S4.

## 3. Results

### 3.1. Naringin Supplementation during the Third Week of Pregnancy Positively Modulate the Males’ Prefrontal Cortex Redox Status on Postnatal Day 7

**Figure 1:**
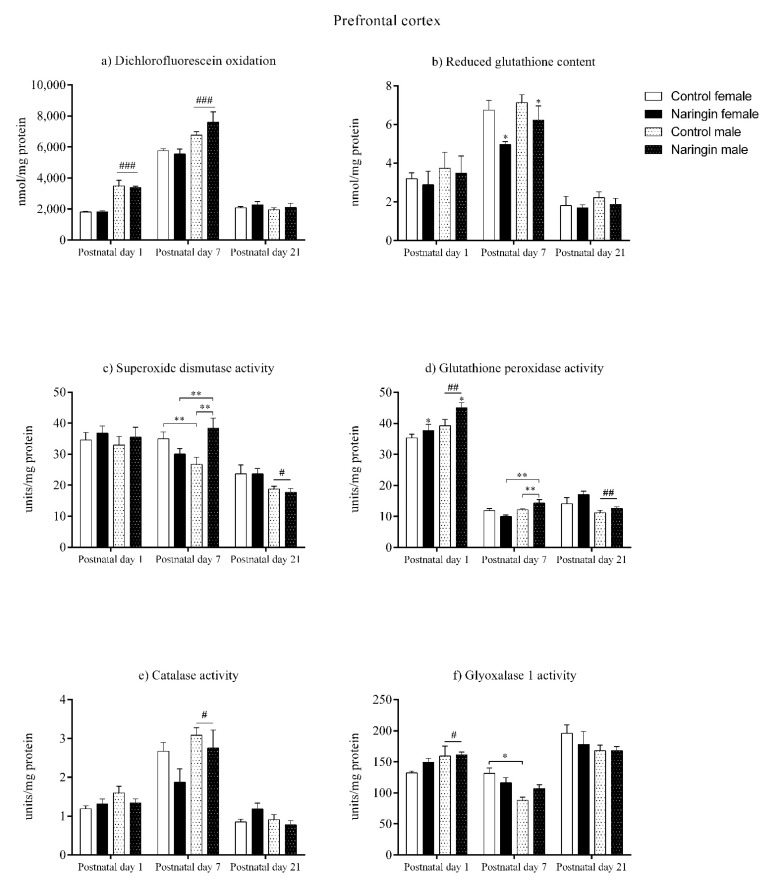
Effects of maternal naringin supplementation during the third week of gestation on the (**a**) dichlorofluorescein oxidation, (**b**) reduced glutathione content, (**c**) superoxide dismutase activity, (**d**) glutathione-peroxidase activity, (**e**) catalase activity, and (**f**) glyoxalase 1 activity in the offspring’s prefrontal cortex on postnatal days 1, 7, and 21. Results are expressed as mean ± S.E.M. Control female *n* = 8, naringin female *n* = 8, control male *n* = 8, naringin male *n* = 6. * *p* < 0.05; ** *p* < 0.01; # *p* < 0.05; ## *p* < 0.01, ### *p* < 0.001 (two-way ANOVA). Asterisks represent the main supplementation effect, and the hashes on bars represent the main sex effect. The asterisks on bars (single bracket) represent the significant comparisons by Sidak’s post-hoc test when interactions occurred.

On postnatal day 1, maternal naringin supplementation during pregnancy induced alterations in the offspring’s prefrontal cortex redox status. As demonstrated in Figure 1d, both male and female pups born to naringin-supplemented dams showed increased GPx activity (supplementation effect, *p* = 0.024), even though no other supplementation-associated alterations were observed. Interestingly, our results also demonstrated sex-related differences suggesting that males had increased oxidative status than females since they showed higher total oxidants level (sex effect, *p* < 0.001), GPx (sex effect, *p* = 0.003), and GLO1 (sex effect, *p* = 0.048) activities (Figure 1a,d,f, respectively). Meanwhile, females had a higher SOD/GPx ratio (sex effect, *p* = 0.029) in the prefrontal cortex on postnatal day 1 (Figure 5a).

Moreover, when analyzing the offspring’s prefrontal cortex redox status on postnatal day 7, there were observed sex-specific alterations induced by maternal naringin supplementation. At this age, both male and female offspring born to supplemented dams had increased GSH content (supplementation effect, *p* = 0.012) in the prefrontal cortex (Figure 1b); however, an interaction between sex and supplementation was observed in the SOD (interaction, *p* = 0.002) and GPx (interaction, *p* = 0.008) activities, demonstrating that male pups born to naringin supplemented rats had increased activities compared to males born to control dams (Figure 1c,d, respectively). Moreover, the naringin males showed higher SOD (interaction, *p* = 0.002) and GPx (interaction, *p* = 0.008) activities when compared to the naringin females. The above supplementation-related alterations were accompanied by sex differences in the prefrontal cortex’s redox status in which females had higher SOD (interaction, *p* = 0.002) and GLO1 (interaction, *p* = 0.026) activities (Figure 1c,f, respectively), and SOD/GPx ratio (sex effect, *p* = 0.038) (Figure 5a) when compared to males, while males displayed increased oxidants content (sex effect, *p* < 0.001) CAT activity (sex effect, *p* = 0.038) when compared to females (Figure 1e).

Interestingly, on postnatal day 21, the total number of supplementation-associated alterations reduced, and no interaction between sex and supplementation was observed. With exception of the SOD/GPx ratio (supplementation effect, *p* = 0.022) (Figure 5a), which was increased in the prefrontal cortex of pups born to naringin-supplemented dams, no other supplementation-related alteration was observed in the redox parameters evaluated in our study (Figure 1). In addition, sex-related differences were found in the SOD (sex effect, *p* = 0.011) and GPx (sex effect, *p* = 0.009) activities, which were higher in female than in male pups in the prefrontal cortex on postnatal day 21 (Figure 1c,d, respectively).

### 3.2. Maternal Naringin Supplementation during Pregnancy Triggers Sex-Independent Redox Alterations in the Offspring’s Hippocampus

**Figure 2:**
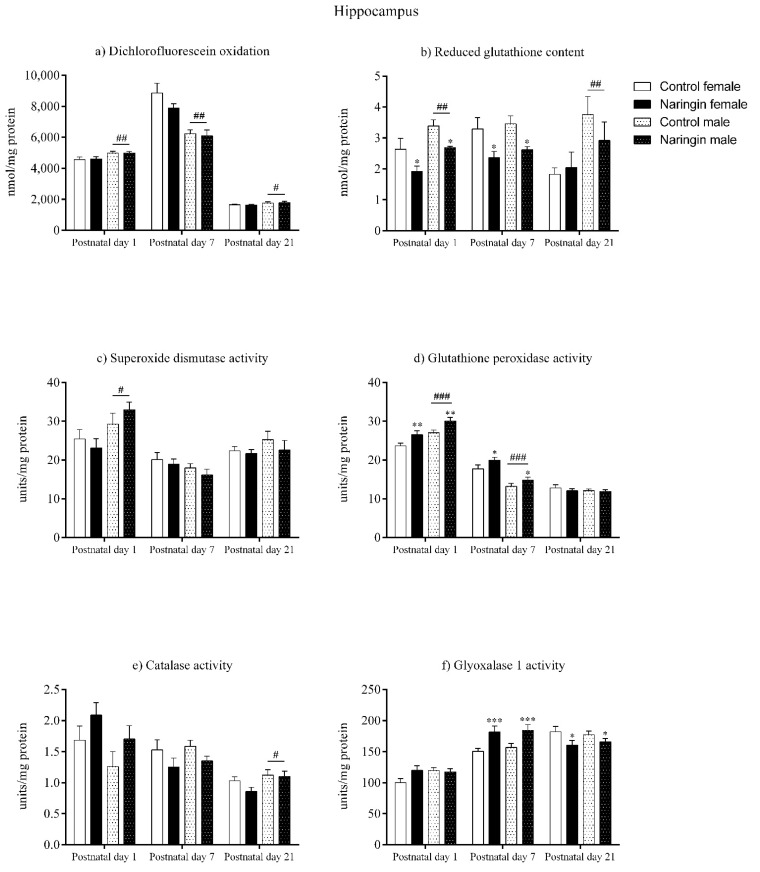
Effects of maternal naringin supplementation during the third week of gestation on the (**a**) dichlorofluorescein oxidation, (**b**) reduced glutathione content, (**c**) superoxide dismutase activity, (**d**) glutathione-peroxidase activity, (**e**) catalase activity, and (**f**) glyoxalase 1 activity in the offspring’s hippocampus on postnatal days 1, 7, and 21. Results are expressed as mean ± S.E.M. Control female *n* = 8, naringin female *n* = 8, control male *n* = 8, naringin male *n* = 6. * *p* < 0.05; ** *p* < 0.01; *** *p* < 0.001; # *p* < 0.05; ## *p* < 0.01; ### *p* < 0.001 (two-way ANOVA). Asterisks represent a main supplementation effect, and the hashes on bars represent a main sex effect. The asterisks on bars (single bracket) represent the significant comparisons by Sidak’s post-hoc test when interactions occurred.

In the hippocampus, maternal naringin supplementation during pregnancy induced alterations in both male and female pups on postnatal day 1. Here, the offspring born to supplemented dams showed reduced GSH content (supplementation effect, *p* = 0.001), which was accompanied by increased GPx activity (supplementation effect, *p* = 0.003), in the hippocampus (Figure 2b,d, respectively), though no other supplementation-associated alteration was observed in the redox status at this postnatal age (Figure 2). In addition, sex-related differences in which males showed higher oxidants (sex effect, *p* = 0.006), GSH content (sex effect, *p* = 0.002), SOD (sex effect, *p* = 0.014), and GPx (sex effect, *p* < 0.001) activities compared to females were also observed in the offspring’s hippocampus on postnatal day 1 (Figure 2a–d, respectively).

Similarly, the alterations elicited by maternal naringin supplementation in the hippocampus’ redox status on postnatal day 1 also persisted on postnatal day 7, as shown in Figure 2. The GSH content (supplementation effect, *p* = 0.002) remained reduced while the GPx activity (supplementation effect, *p* = 0.026) was still increased on postnatal day 7 (Figure 2b,d, respectively), in addition to other supplementation-related effects that were observed in the GLO1 activity (supplementation effect, *p* < 0.001), which increased (Figure 2f), and in the SOD/GPx ratio (supplementation effect, *p* = 0.013), which reduced in the offspring born to supplemented rats (Figure 5b). The above alterations were accompanied by sex-related differences in the total oxidants (sex effect, *p* < 0.001) and GPx activity (sex effect, *p* < 0.001), that were higher in females (Figure 2a,d, respectively), and in the SOD/Gpx ratio (sex effect, *p* = 0.005), which was higher in male pups (Figure 5b).

Although on postnatal day 7 the pups’ hippocampal redox status was highly affected by maternal naringin supplementation, most of these alterations did not persist up to postnatal day 21. While the previous alterations found in the GSH content, GPx activity (Figure 2b,d, respectively) and in the SOD/GPx ratio (Figure 5b) did not show any significant difference on postnatal day 21, the GLO1 activity (supplementation effect, *p* = 0.039) reduced in the offspring born to naringin-supplemented dams, which contrasts from the increased GLO1 activity previously detected on postnatal day 7 (Figure 2f). Besides, sex-related differences were observed on postnatal day 21 in which males had higher oxidants level (sex effect, *p* = 0.045), GSH content (sex effect, *p* = 0.006), and CAT activity (sex effect, *p* = 0.037) in the hippocampus when compared to the female pups (Figure 2a,b,e, respectively).

### 3.3. Naringin Supplementation during Pregnancy Induces a Pro-Oxidative Shift in the Male Offspring’s Striatum on Postnatal Day 7

**Figure 3:**
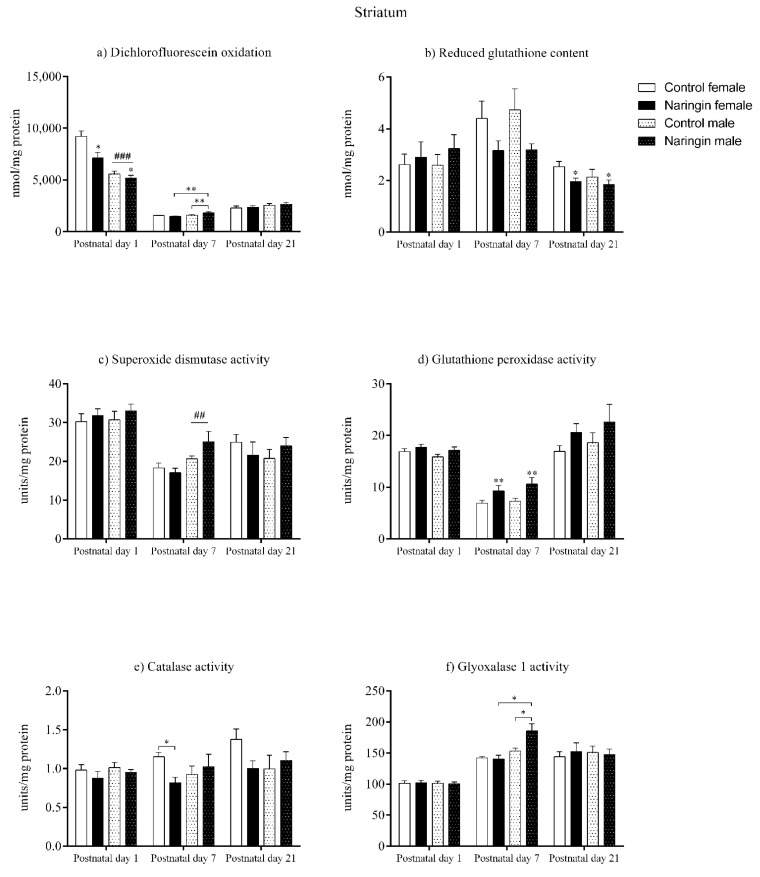
Effects of maternal naringin supplementation during the third week of gestation on the (**a**) dichlorofluorescein oxidation, (**b**) reduced glutathione content, (**c**) superoxide dismutase activity, (**d**) glutathione-peroxidase activity, (**e**) catalase activity, and (**f**) glyoxalase 1 activity in the offspring’s striatum on postnatal day 1, 7 and 21. Results are expressed as mean ± S.E.M. Control female *n* = 8, naringin female *n* = 8, control male *n* = 8, naringin male *n* = 6. * *p* < 0.05; ** *p* < 0.01; ## *p* < 0.01; ### *p* < 0.001 (two-way ANOVA). Asterisks represent a main supplementation effect, and the hashes on bars represent a main sex effect. The asterisks on bars (single bracket) represent the significant comparisons by Sidak’s post-hoc test when interactions occurred.

On postnatal day 1, the offspring born to naringin supplemented rats showed reduced total oxidants content (supplementation effect, *p* < 0.001) in the striatum (Figure 3a), even though no other supplementation-related alterations in the redox status were found at this postnatal age (Figure 3). Such alteration was also accompanied by a sex-associated difference in which females demonstrated higher oxidants content compared to males (sex effect, *p* = 0.007) also on postnatal day 1 (Figure 3a).

Moreover, on postnatal day 7, our results showed that maternal naringin supplementation during pregnancy affected the pups’ redox status in a sex-specific manner in the striatum. An interaction between supplementation and sex was observed in the total oxidants content (interaction, *p* = 0.008) in which naringin male pups showed increased oxidants level compared to control males, even though the same effects were not observed in the female pups’ striatum (Figure 3a). Additionally, the naringin male pups had higher oxidants content when compared to the naringin female pups (interaction, *p* = 0.008). Similarly, the GLO1 and CAT activities also showed interactions between supplementation and sex (interaction, *p* = 0.020; *p* = 0.049; respectively), in which male pups born to naringin-supplemented rats had increased GLO1 activity compared to control males and naringin females (Figure 3f), and females born to supplemented dams showed decreased CAT activity compared to control female pups (Figure 3e).

In addition to the interactions between supplementation and sex detected on postnatal day 7, we also observed other supplementation and sex-related effects in the offspring’s striatum at this postnatal age. As shown in Figure 3d and 5, the maternal naringin supplementation during pregnancy also induced an increase in the GPx activity (supplementation effect, *p* = 0.003), which probably led to a reduction in the SOD/GPx ratio (supplementation effect, *p* = 0.001) in both male and female pups (Figure 5c). Moreover, a sex-associated difference was observed in which males showed higher SOD activity (sex effect, *p* = 0.002) compared to the female pups (Figure 3c).

Despite naringin supplementation induced several alterations in the redox status in the offspring’s striatum on postnatal day 7, this same pattern was not observed on postnatal day 21, since the only effect elicited by the maternal supplementation was the reduction in the GSH content (supplementation effect, *p* = 0.042), as shown in Figure 3b. Besides, no sex-related differences were observed between male and female pups on postnatal day 21.

### 3.4. Maternal Naringin Supplementation during the Third Week of Pregnancy Induces a Pro-Oxidative Milieu in the Female Pups’ Cerebellum on Postnatal Day 7

**Figure 4:**
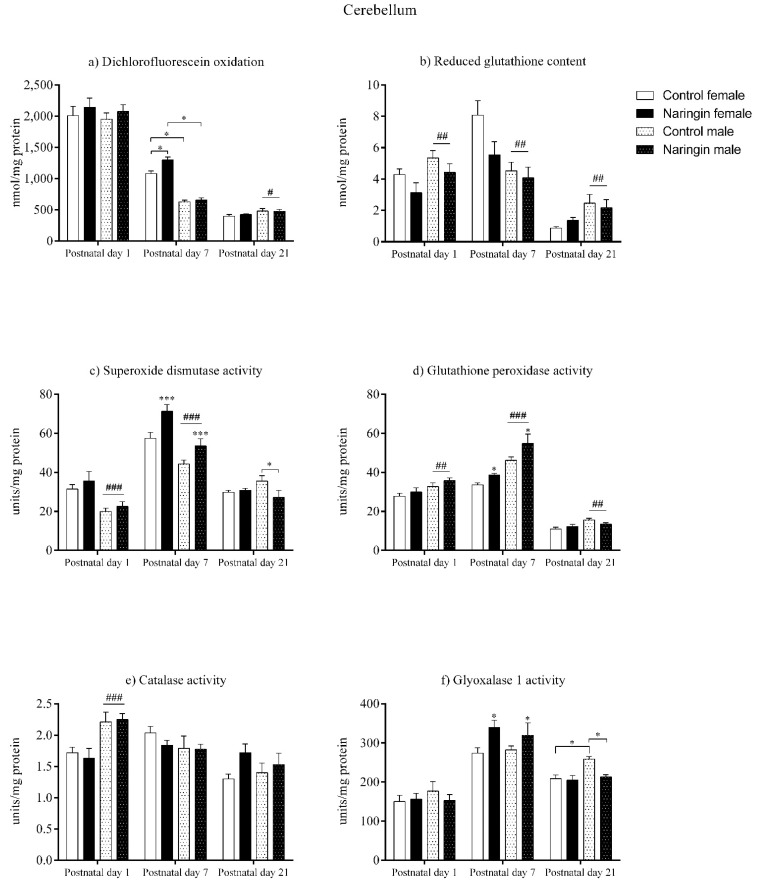
Effects of maternal naringin supplementation during the third week of gestation on the (**a**) dichlorofluorescein oxidation, (**b**) reduced glutathione content, (**c**) superoxide dismutase activity, (**d**) glutathione-peroxidase activity, (**e**) catalase activity, and (**f**) glyoxalase 1 activity in the offspring’s cerebellum on postnatal days 1, 7, and 21. Results are expressed as mean ± S.E.M. Control female *n* = 8, naringin female *n* = 8, control male *n* = 8, naringin male *n* = 6. * *p* < 0.05; *** *p* < 0.001 # *p* < 0.05; ## *p* < 0.01; ### *p* < 0.001 (two-way ANOVA). Asterisks represent a main supplementation effect, and the hashes on bars represent a main sex effect. The asterisks on bars (single bracket) represent the significant comparisons by Sidak’s post-hoc test when interactions occurred.

**Figure 5:**
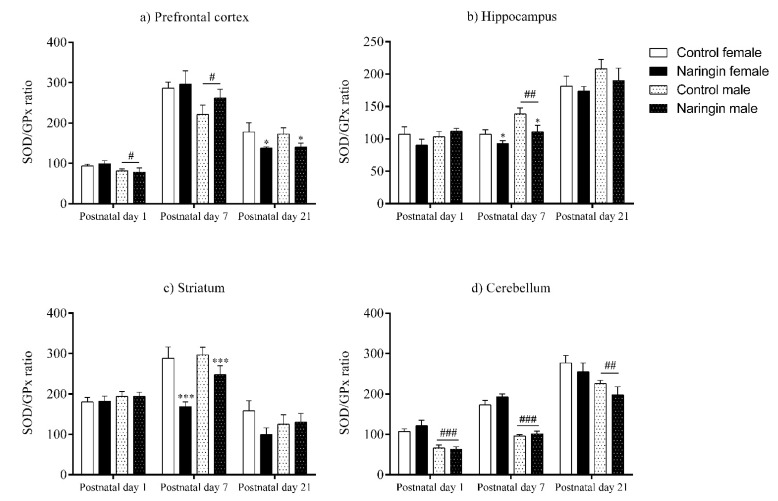
Effects of maternal naringin supplementation during the third week of gestation on the SOD/GPx ratio in the offspring’s (**a**) prefrontal cortex, (**b**) hippocampus, (**c**) striatum, and (**d**) cerebellum on postnatal days 1, 7, and 21. Results are expressed as mean ± S.E.M. Control female *n* = 8, naringin female *n* = 8, control male *n* = 8, naringin male *n* = 6. * *p* < 0.05; *** *p* < 0.001 # *p* < 0.05; ## *p* < 0.01; ### *p* < 0.001 (two-way ANOVA). Asterisks represent a main supplementation effect, and the hashes on bars represent a main sex effect. The asterisks on bars (single bracket) represent the significant comparisons by Sidak’s post-hoc test when interactions occurred.

As demonstrated by our findings, the maternal naringin supplementation during pregnancy did not induce alterations in the offspring’s cerebellum redox status on postnatal day 1, as shown in Figure 4. However, sex-related differences were observed at this postnatal age, in which males showed higher GSH levels (sex effect, *p* = 0.003), GPx (sex effect, *p* = 0.004), and CAT (sex effect, *p* < 0.001) activities (Figure 4b,d,e, respectively), while females displayed higher SOD (sex effect, *p* < 0.001) activity (Figure 4c) and SOD/GPx ratio (sex effect, *p* < 0.001) in the cerebellum (Figure 5c).

On postnatal day 7, in contrast to the findings observed on postnatal day 1, the offspring were affected by maternal naringin supplementation, though the effect occurred in a sex-specific manner. Both female and male pups born to naringin supplemented rats demonstrated increased SOD (supplementation effect, *p* = 0.001), GPx (supplementation effect, *p* = 0.012), and GLO1 (supplementation effect, *p* = 0.015) activities (Figure 4c,d,f, respectively) in the cerebellum one week after birth, and such enzymatic alterations were accompanied by a significant interaction between supplementation and sex in the total oxidants level (interaction, *p* = 0.021). This effect showed that naringin females had increased oxidants compared to the control female pups, but naringin males did not show any alteration regarding the same variable (Figure 4a). Moreover, the naringin females displayed higher oxidants when compared to the naringin males (interaction, *p* = 0.021).

Moreover, such interaction also demonstrated that the control females had higher oxidants content in the cerebellum when compared to the control males on postnatal day 7 (Figure 4a). This difference was followed by other sex-related effects in which females showed higher GSH content (sex effect, *p* = 0.002), SOD activity (sex effect, *p* < 0.001) (Figure 4b,c, respectively), and SOD/GPx ratio (sex effect, *p* < 0.001) (Figure 5c), while males showed a higher GPx activity (sex effect, *p* < 0.001), demonstrating that on postnatal day 7 the female offspring possessed a higher oxidative status than males of the same age (Figure 4d).

Additionally, the redox alterations induced by maternal naringin supplementation in the female’s cerebellum on postnatal day 7 did not persist up to postnatal day 21, since we did not observe any supplementation-associated effect at this age, as demonstrated in Figure 4. In contrast, the male pups showed two significant interactions between supplementation and sex in the SOD (interaction, *p* = 0.035) and GLO1 (interaction, *p* = 0.031) activities on postnatal day 21, in which male pups born to naringin-supplemented rats showed decreased activities of both enzymes when compared to the control males of the same postnatal age (Figure 4c,f, respectively).

Lastly, the sex-related differences found in the offspring’s cerebellum on postnatal day 21 showed that the male pups, differently from postnatal day 7, have increased redox status than the female pups from the same postnatal age (Figure 4). Here, males showed higher total oxidants level (sex effect, *p* = 0.022), GSH content (sex effect, *p* = 0.004), GPx (sex effect, *p* = 0.006), and GLO1 (interaction, *p* = 0.031) activities (Figure 4a,b,d,f, respectively), while the female pups had higher SOD/GPx ratio (sex effect, *p* = 0.006) at this postnatal age (Figure 5c).

## 4. Discussion

Although polyphenols can induce beneficial effects when supplemented to adult rodents, recent evidence has been demonstrating that polyphenol supplementation can induce detrimental effects if consumed during sensitive periods of development, such as pregnancy, which can affect the fetal intrauterine development and lead to physiological alterations during the postnatal life [21,48,49,50]. Therefore, since naringin is a polyphenol able to modulate the redox status, we hypothesized that maternal naringin supplementation during the third week of pregnancy can induce alterations in the offspring’s brain redox status during postnatal life.

Considering the total number of alterations found in our model of maternal naringin supplementation, we notice that they are not proportionally distributed among the brain regions and the postnatal ages evaluated here. Such observation demonstrates that the effects induced by maternal naringin supplementation during the third week of pregnancy are still active during offspring’s postnatal development, mainly on postnatal day 7. However, most of the redox alterations observed in the younger pups did not persist up to postnatal day 21, which suggests that they are transitory and did not persist along with the offspring’s brain development. Similarly, AUGUST et al. (2018) also observed the same pattern in which maternal supplementation with naringenin, the aglycone form of naringin, did not induce alterations in the offspring’s brain redox status on postnatal day 21, even though only male pups were evaluated [51].

Moreover, on postnatal day 1, the maternal naringin supplementation during the third week of pregnancy induced a positive modulation in the offspring’s brain redox status that occurred in a region-specific manner. As shown in Table 1, the reduced total oxidants content in the pups’ striatum was not accompanied by alterations in the antioxidant enzymes activity and GSH level, even though the hippocampus and the prefrontal cortex showed the opposite pattern: alterations in the GPx and GSH, and no changes in the oxidants level with 1 week of maternal naringin supplementation. The total oxidants content is commonly assessed by the DCFH, and together with the antioxidant enzymes activity and GSH levels, they can demonstrate the redox status of a cell or tissue, thereby suggesting that in our model, with exception of the cerebellum, the maternal naringin supplementation triggered an antioxidant milieu in the pup’s brain regions on postnatal day 1 [52]. Accordingly, evidence elsewhere has also demonstrated that naringin supplementation can positively modulate the redox status in adult rat’s brains, improving antioxidants defenses and reducing the oxidants content, though it occurred with more than one week of naringin intake [34,53,54].

Interestingly, even though naringin supplementation during pregnancy induced an antioxidant effect in the pup’s brain on postnatal day 1, such alteration did not predominantly occur on postnatal day 7. In the cerebellum and striatum, naringin positively regulated most of the antioxidant enzymes in the male and female offspring; however, both brain regions also displayed a sex-dependent pro-oxidant effect demonstrated by the higher oxidants content. Such increase is in contrast with most published data demonstrating that naringin promotes neuroprotection by reducing oxidants and increasing antioxidant defenses when supplemented to adult rats submitted to different models of cognitive dysfunction [33,55,56,57,58,59]. However, it is also important to consider that in our model naringin alone increased the total oxidants content, a pro-oxidative property of flavonoids that was already demonstrated in vitro in a dose-dependent manner [60,61]. Thereby, such prooxidative milieu may have induced the increase in the antioxidant enzyme activities as a compensatory mechanism in the females’ cerebellum and the males’ striatum, which suggests that regarding these brain regions, female and male offspring were negatively affected by naringin consumption during gestation [62].

Additionally, even though naringin induced a prooxidative effect in the pups’ cerebellum and striatum, such alterations were not observed in the prefrontal cortex and hippocampus on postnatal 7 since these brain regions exhibited a positive modulation of the redox status. As shown in Table 1, maternal naringin supplementation reduced the GSH content in the offspring’s prefrontal cortex in both sexes, but the male pups also displayed higher SOD and GPx activities, which has been demonstrated in adult male rats’ cerebral cortex in a dose-dependent manner [54]. Similarly, the pups’ hippocampal antioxidant enzymes were positively modulated while the GSH content was reduced on postnatal day 7. The GSH is the main non-enzymatic antioxidant in the cells that can directly react with reactive species or participate in enzymatic pathways, which suggests that such lower GSH levels might have occurred due to the positive regulation exerted by naringin in the GPx and GLO1 activities in the hippocampus from naringin-exposed pups [42,45,63].

Moreover, further analyses of the brain redox status on postnatal day 21 demonstrated that the naringin-induced alterations observed on postnatal day 7 were transient and did not persist up to weaning. Even though most of the brain regions displayed negative modulations of the GSH, GLO1, and SOD/GPx ratio in both sexes, the cerebellum showed sex-dependent effects in which only males had lower SOD and GLO1 activities. Such alterations observed on postnatal day 21 demonstrate that naringin negatively modulated such parameters, which contrasts with data showing that naringenin supplementation to pregnant rats during gestation did not induce redox alterations in the male pups’ cerebellum and hypothalamus, though the naringenin dose used was 50 mg/kg/day [51].

Despite the alterations observed in our model, the underlying mechanisms by which naringin or flavonoid supplementation during pregnancy can modify fetal redox homeostasis remain largely unknown. It is well established that the redox signaling is essential during neurodevelopment since the transition from neural stem cells (NSC) to a neuron involves a metabolic shift from a glycolytic to a more oxidative based metabolism, which consequently increases the amount of mitochondrial-derived reactive oxygen species (ROS) [64,65]. Moreover, other sources of ROS, such as the NADPH-oxidase enzyme family, have also been proposed to play particularly important roles in inducing the neurogenic process [66,67,68]. Consequently, such an increase in the ROS induces the activation of several signaling cascades involved in the commitment of the NSC and its differentiation into a fully grown neuron [68,69,70,71].

Although the transient increase in ROS is essential to induce the commitment of the NSC, such a process needs to be tightly regulated to maintain a physiological and tolerable intracellular level of reactive species [72]. On one hand, persistent and particularly high amounts of ROS, which exceed a physiologically acceptable range, can induce oxidative stress, hinder axonal growth, and impair neurodevelopment, while on the other hand, low ROS amounts have been linked to the maintenance of the quiescent state of the NSC, which reduces its differentiation into neurons [67,73,74].

In addition, the neurogenesis in the developing rat brain can also be affected by alterations in the neuronal redox status. Both the deletion of the PR domain containing 16 (PRDM16) and the mitochondrial uncoupling protein 2 (UCP) in knockout mice during embryonic development strongly increased ROS levels, which were associated with abnormal cortical lawyer thickness and development [75,76]. Other cortical and hippocampal neurodevelopmental alterations related to increased ROS content have also been reported by a similar study during embryonic and adult life, which resulted in abnormal motor behavior along with memory and learning defects [77]. However, the abnormal decrease in the ROS levels during brain development has also been associated with histopathological abnormalities in the neonatal rat’s cerebellum, which were accompanied by altered motor behavior [78]. Such findings demonstrate the important role that ROS plays as signaling molecules mediating the developing brain neurogenesis. More importantly, they also suggest that in healthy animals, neither a positive nor a negative modulation of the brain’s redox status is beneficial to neurodevelopment, since both high and low amounts of reactive species have been shown to alter neurogenesis and induce anatomical and behavioral defects.

Since flavonoids are redox active molecules, they possess a scavenger activity and can directly react with ROS, as well as modulate cell signaling pathways that regulate the expression of several cellular components of the redox network [79,80,81]. It has been demonstrated that flavonoids and their metabolites have already been detected in the breast milk, amniotic fluid, and rat fetuses, suggesting they can cross the placental barrier and exert their redox regulation directly in the fetal tissues, and consequently modulating the fetal redox status, which can possibly interfere during normal neurodevelopment [82,83,84]. Moreover, maternal resveratrol supplementation has been shown to modify the methylation levels of the factor nuclear kappa β (NF-κβ) promoter region and the gene expression of the peroxisome proliferator-activated receptor gamma coactivator 1-alpha (*Pgc-1α*) and heme oxygenase 1 (*Hmox1*) genes in the offspring’s hippocampus in a model of accelerated senescence during adult life, suggesting that epigenetic modifications may underlie the metabolic effects induced by the high flavonoid intake during gestation [85]. However, as demonstrated by our findings, the alterations induced by maternal naringin supplementation in the offspring’s redox status differ according to the brain region, so a given underlying mechanism may be differentially affected according to the developmental stage of each brain region during the period in which the supplementation occurred.

Although the rat and human trajectories of CNS development do not directly correspond, they can be relatively compared when considering the processes that occur during brain development, such as neurogenesis, myelination, synaptogenesis, and synaptic pruning [35,36,86]. As previously discussed, most of the alterations were detected on postnatal day 7, which relatively corresponds to a newborn human when considering brain development [35,36,86]. Moreover, as demonstrated by our findings, the brain regions were differentially affected by maternal naringin supplementation, which might be partially explained by their temporal differences in the developmental processes mentioned above, since such differences also mean distinct neuronal needs and ROS tolerance [35,36,72,86]. However, whether maternal naringin supplementation during pregnancy induces anatomical and behavioral alterations in the offspring, remains a topic for future investigation.

Lastly, as shown in the introduction, the anti-inflammatory and antioxidant properties of polyphenols induced alterations in the closure dynamics of the fetal *ductus arteriosus* when consumed in high amounts during the third trimester of pregnancy, which is the critical window of development of the fetus’ ductus [27,28,87]. Similarly, we have demonstrated that maternal naringin supplementation during the third week of gestation induced redox alterations in the offspring’s brain, mainly on postnatal day 7, which promoted a sex-dependent pro-oxidative milieu in the cerebellum and striatum at this postnatal age. Although such alterations did not persist up to weaning, they might be detrimental to brain development, since evidence demonstrates that the neonatal brain is more susceptible to oxidative stress, particularly when further considering that redox alterations during the neonatal period can modify the NSC commitment course, which impairs the neurogenic process and further leads to anatomical and behavioral defects [75,76,78,88,89,90,91].

## 5. Conclusions

We demonstrated for the first time that naringin supplementation during the third week of pregnancy induced region and sex-specific redox alterations in the offspring’s brain during postnatal development. Even though maternal naringin consumption positively modulated the pups’ brain redox status on postnatal day 1, such effect was not observed on postnatal day 7, in which a pro-oxidative milieu was induced in a sex-dependent manner, thereby suggesting that females and males were specifically affected by the maternal naringin consumption in the cerebellum and striatum, respectively. Differently, the prefrontal cortex and the hippocampus’ redox status was positively modulated on postnatal 7. Nevertheless, though naringin induces either a positive or a negative modulation in the offspring’s brain redox status, both situations can be detrimental to the NSC commitment process and neurodevelopment. Therefore, future research should focus to unveil the mechanisms underlying the redox alterations induced by maternal naringin supplementation and if such alterations can induce anatomical and behavioral defects related to each of the offspring’s brain regions.

## Figures and Tables

**Table 1:** Summary of the alterations induced by maternal naringin supplementation during the third week of gestation in the offspring’s brain redox status during postnatal development.

	Prefrontal Cortex		Hippocampus		Striatum		Cerebellum	
	Female	Male	Female	Male	Female	Male	Female	Male
Postnatal day 1	↑ GPx activity	↑ GPx activity	↓ GSH content ↑ GPx activity	↓ GSH content ↑ GPx activity	↓ Total oxidants content	↓ Total oxidants content		
Postnatal day 7	↓ GSH content	↓ GSH content ↑ SOD activity ↑ GPx activity	↓ GSH content ↑ GPx activity ↑ GLO1 activity ↓ SOD/GPx ratio	↓ GSH content ↑ GPx activity ↑ GLO1 activity ↓ SOD/GPx ratio	↑ GPx activity ↓ CAT activity ↓ SOD/GPx ratio	↑ Total oxidants content ↑ GPx activity ↑ GLO1 activity ↓ SOD/GPx ratio	↑ Total oxidants content ↑ SOD activity ↑ GPx activity ↑ GLO1 activity	↑ SOD activity ↑ GPx activity ↑ GLO1 activity
Postnatal day 21	↓ SOD/GPx ratio	↓ SOD/GPx ratio	↓ GLO1 activity	↓ GLO1 activity	↓ GSH content	↓ GSH content		↓ SOD activity ↓ GLO1 activity

↓: means the given parameter was significantly lower in the naringin pups compared to the control pups, ↑: means the given parameter was significantly higher in the naringin pups compared to the control pups.

## Data Availability

The data presented in this study are available in the Supplementary Tables S1–S4.

## Supplementary Materials

The following are available online at https://www.mdpi.com/article/10.3390/ijerph18094805/s1, Table S1: Statistical data from the biochemical analyses performed in the offspring’s prefrontal cortex. Table S2: Statistical data from the biochemical analyses performed in the offspring’s hippocampus. Table S3: Statistical data from the biochemical analyses performed in the offspring’s striatum. Table S4: Statistical data from the biochemical analyses performed in the offspring’s cerebellum.

## References

[1] Chen R., Qi Q.L., Wang M.T., Li Q.Y. (2016). Therapeutic potential of naringin: An overview. Pharm. Biol..

[2] Viswanatha G.L., Shylaja H., Moolemath Y. (2017). The beneficial role of Naringin- a citrus bioflavonoid, against oxidative stress-induced neurobehavioral disorders and cognitive dysfunction in rodents: A systematic review and meta-analysis. Biomed. Pharmacother..

[3] Wang D., Gao K., Li X., Shen X., Zhang X., Ma C., Qin C., Zhang L. (2012). Long-term naringin consumption reverses a glucose uptake defect and improves cognitive deficits in a mouse model of Alzheimer’s disease. Pharmacol. Biochem. Behav..

[4] Chtourou Y., Gargouri B., Kebieche M., Fetoui H. (2015). Naringin Abrogates Cisplatin-Induced Cognitive Deficits and Cholinergic Dysfunction Through the Down-Regulation of AChE Expression and iNOS Signaling Pathways in Hippocampus of Aged Rats. J. Mol. Neurosci..

[5] Golechha M., Sarangal V., Bhatia J., Chaudhry U., Saluja D., Arya D.S. (2014). Naringin ameliorates pentylenetetrazol-induced seizures and associated oxidative stress, inflammation, and cognitive impairment in rats: Possible mechanisms of neuroprotection. Epilepsy Behav..

[6] Dow C.A., Going S.B., Chow H.H.S., Patil B.S., Thomson C.A. (2012). The effects of daily consumption of grapefruit on body weight, lipids, and blood pressure in healthy, overweight adults. Metabolism.

[7] Jung U.J., Kim H.J., Lee J.S., Lee M.K., Kim H.O., Park E.J., Kim H.K., Jeong T.S., Choi M.S. (2003). Naringin supplementation lowers plasma lipids and enhances erythrocyte antioxidant enzyme activities in hypercholesterolemic subjects. Clin. Nutr..

[8] Aptekmann N.P., Cesar T.B. (2013). Long-term orange juice consumption is associated with low LDL-cholesterol and apolipoprotein B in normal and moderately hypercholesterolemic subjects. Lipids Health Dis..

[9] Kurowska E.M., Spence J.D., Jordan J., Wetmore S., Freeman D.J., Piche L.A., Serratore P. (2000). HDL-cholesterol-raising effect of orange juice in subjects with hypercholesterolemia. Am. J. Clin. Nutr..

[10] Snyder F.J., Dundas M.L., Kirkpatrick C., Neill K.S. (2009). Use and safety perceptions regarding herbal supplements: A study of older persons in Southeast Idaho. J. Nutr. Elder..

[11] Kirkpatrick C.F., Page R.M., Hayward K.S. (2006). Nonvitamin, nonmineral supplement use and beliefs about safety and efficacy among rural older adults in southeast and south central Idaho. J. Nutr. Elder..

[12] Read M.H., Klomp S., Mather D., Todd S. (2002). Use of Herbal Supplements Reported by Older Adults in Congregate Meal Sites. Top. Clin. Nutr..

[13] Harnack L.J., DeRosier K.L., Rydell S.A. (2003). Results of a population-based survey of adults’ attitudes and beliefs about herbal products. J. Am. Pharm. Assoc..

[14] Holst L., Wright D., Haavik S., Nordeng H. (2009). Remedies During Pregnancy. J. Altern. Complement. Med..

[15] Forster D.A., Denning A., Wills G., Bolger M., McCarthy E. (2006). Herbal medicine use during pregnancy in a group of Australian women. BMC Pregnancy Childbirth.

[16] Kennedy D.A., Lupattelli A., Koren G., Nordeng H. (2013). Herbal medicine use in pregnancy: Results of a multinational study. BMC Complement. Altern. Med..

[17] Nordeng H., Havnen G.C. (2004). Use of herbal drugs in pregnancy: A survey among 400 Norwegian women. Pharmacoepidemiol. Drug Saf..

[18] Skibola C.F., Smith M.T. (2000). Potential health impacts of excessive flavonoid intake. Free Radic. Biol. Med..

[19] Mennen L.I., Walker R., Bennetau-Pelissero C., Scalbert A. (2005). Risks and safety of polyphenol consumption. Am. J. Clin. Nutr..

[20] Suzuki K. (2018). The developing world of DOHaD. J. Dev. Orig. Health Dis..

[21] Hachul A.C.L., Boldarine V.T., Neto N.I.P., Moreno M.F., Carvalho P.O., Sawaya A.C.H.F., Ribeiro E.B., Oller do Nascimento C.M., Oyama L.M. (2018). Effect of the consumption of green tea extract during pregnancy and lactation on metabolism of mothers and 28d-old offspring. Sci. Rep..

[22] Del Bas J.M., Crescenti A., Arola-Arnal A., Oms-Oliu G., Arola L., Caimari A. (2015). Intake of grape procyanidins during gestation and lactation impairs reverse cholesterol transport and increases atherogenic risk indexes in adult offspring. J. Nutr. Biochem..

[23] Vanhees K., van Schooten F.J., van Waalwijk van Doorn-Khosrovani S.B., van Helden S., Munnia A., Peluso M., Briedé J.J., Haenen G.R.M.M., Godschalk R.W.L. (2013). Intrauterine exposure to flavonoids modifies antioxidant status at adulthood and decreases oxidative stress-induced DNA damage. Free Radic. Biol. Med..

[24] Hilger D.K., Wohlenberg M.F., Schaffer T.K., de Machado F.S., Gonçalves L.K., Bortolato G., Dani G., Rodrigues A., Funchal C., Dani C. (2015). Purple Grape Juice, an Important Flavonoids Source, Influence in Biochemical Parameters in Offspring of Wistar Rats. Food Nutr. Sci..

[25] Schaffer T.K., Wohlenberg M.F., de Souza Machado F., Bortolato G., Marinho J.P., da Silva Medeiros N., Mello A., Agostini F., Gerson S., Funchal C. (2019). Chronic consumption of purple grape juice in gestational-lactation and post lactation promotes anxiolity effect and antioxidant defense improvement in brain from Wistar male offsprings. J. Nutr. Intermed. Metab..

[26] Barenys M., Gassmann K., Baksmeier C., Heinz S., Reverte I., Schmuck M., Temme T., Bendt F., Zschauer T.-C., Rockel T.D. (2017). Epigallocatechin gallate (EGCG) inhibits adhesion and migration of neural progenitor cells in vitro. Arch. Toxicol..

[27] Zielinsky P., Piccoli A.L., Manica J.L., Nicoloso L.H., Menezes H., Busato A., Moraes M.R., Silva J., Bender L., Pizzato P. (2010). Maternal consumption of polyphenol-rich foods in late pregnancy and fetal ductus arteriosus flow dynamics. J. Perinatol..

[28] Bubols G.B., Zielinsky P., Piccoli A.L., Nicoloso L.H., Vian I., Moro A.M., Charão M.F., Brucker N., Bulcão R.P., Nascimento S.N. (2014). Nitric oxide and reactive species are modulated in the polyphenol-induced ductus arteriosus constriction in pregnant sheep. Prenat. Diagn..

[29] Zielinsky P., Busato S. (2013). Prenatal effects of maternal consumption of polyphenol-rich foods in late pregnancy upon fetal ductus arteriosus. Birth Defects Res. Part C Embryo Today Rev..

[30] Castillo J., Benavente O., del Rio J.A. (1992). Naringin and Neohesperidin Levels during Development of Leaves, Flower Buds, and Fruits of Citrus aurantium. Plant Physiol..

[31] Yusof S., Ghazali H.M., King G.S. (1990). Naringin content in local citrus fruits. Food Chem..

[32] Stone V., Crestani M.S., Saccomori A.B., Mariño dal Magro B., Maurmann R.M., August P.M., dos Santos B.G., Klein C.P., Hackenhaar F.S., da Silveira Benfato M. (2019). Gestational caloric restriction improves redox homeostasis parameters in the brain of Wistar rats: A screening from birth to adulthood. J. Nutr. Biochem..

[33] Gaur V., Aggarwal A., Kumar A. (2009). Protective effect of naringin against ischemic reperfusion cerebral injury: Possible neurobehavioral, biochemical and cellular alterations in rat brain. Eur. J. Pharmacol..

[34] Chtourou Y., Aouey B., Kebieche M., Fetoui H. (2015). Protective role of naringin against cisplatin induced oxidative stress, inflammatory response and apoptosis in rat striatum via suppressing ROS-mediated NF-κB and P53 signaling pathways. Chem. Biol. Interact..

[35] Rice D., Barone S. (2000). Critical Periods of Vulnerability for the Developing Nervous System: Evidence from Humans and Animal Models. Environ. Health Perspect..

[36] Andersen S.L. (2003). Trajectories of brain development: Point of vulnerability or window of opportunity?. Neurosci. Biobehav. Rev..

[37] Reemst K., Noctor S.C., Lucassen P.J., Hol E.M. (2016). The indispensable roles of microglia and astrocytes during brain development. Front. Hum. Neurosci..

[38] Kupper L.L. (2014). Litter Effect. Wiley StatsRef: Statistics Reference Online.

[39] LeBel C.P., Ischiropoulos H., Bondy S.C. (1992). Evaluation of the Probe 2′,7′-Dichlorofluorescin as an Indicator of Reactive Oxygen Species Formation and Oxidative Stress. Chem. Res. Toxicol..

[40] Conceição E.P.S., Moura E.G., Carvalho J.C., Oliveira E., Lisboa P.C. (2015). Early redox imbalance is associated with liver dysfunction at weaning in overfed rats. J. Physiol..

[41] Misra H.P., Fridovich I. (1972). The role of superoxide anion in the autoxidation of epinephrine and a simple assay for superoxide dismutase. J. Biol. Chem..

[42] Wendel A. (1981). Glutathione Peroxidase. Methods Enzymol..

[43] Aebi H. (1984). [13] Catalase in Vitro. Methods Enzymol..

[44] Thornalley P.J., Tisdale M.J. (1988). Inhibition of proliferation of human promyelocytic leukaemia HL60 cells by S-d-lactoylglutathione in vitro. Leuk. Res..

[45] Browne R.W., Armstrong D. (1998). Reduced glutathione and glutathione disulfide. Methods Mol. Biol..

[46] Lowry O.H., Rosebrough N.J., Farr A.L., Randall R.J. (1951). Protein measurement with the Folin phenol reagent. J. Biol. Chem..

[47] Peterson G.L. (1977). A simplification of the protein assay method of Lowry et al. which is more generally applicable. Anal. Biochem..

[48] Caimari A., Mariné-Casadó R., Boqué N., Crescenti A., Arola L., del Bas J.M. (2017). Maternal intake of grape seed procyanidins during lactation induces insulin resistance and an adiponectin resistance-like phenotype in rat offspring. Sci. Rep..

[49] Bałan B.J., Skopińska-Różewska E., Skopiński P., Zdanowski R., Leśniak M., Kiepura A., Lewicki S. (2017). Morphometric abnormalities in the spleen of the progeny of mice fed epigallocatechin during gestation and nursing. Pol. J. Vet. Sci..

[50] Hachul A.C.L., Boldarine V.T., Neto N.I.P., Moreno M.F., Ribeiro E.B., do Nascimento O.C.M., Oyama L.M. (2018). Maternal consumption of green tea extract during pregnancy and lactation alters offspring’s metabolism in rats. PLoS ONE.

[51] August P.M., Maurmann R.M., Saccomori A.B., Scortegagna M.C., Flores E.B., Klein C.P., dos Santos B.G., Stone V., Dal Magro B.M., Cristhian L. (2018). Effect of maternal antioxidant supplementation and/or exercise practice during pregnancy on postnatal overnutrition induced by litter size reduction: Brain redox homeostasis at weaning. Int. J. Dev. Neurosci..

[52] Eruslanov E., Armstrong D. (2010). Advanced Protocols in Oxidative Stress I.

[53] Kumar A., Prakash A., Dogra S. (2010). Naringin alleviates cognitive impairment, mitochondrial dysfunction and oxidative stress induced by d-galactose in mice. Food Chem. Toxicol..

[54] Sachdeva A.K., Chopra K. (2015). Naringin mitigate okadaic acid-induced cognitive impairment in an experimental paradigm of Alzheimer’s disease. J. Funct. Foods.

[55] Kumar P., Kumar A. (2010). Protective effect of hesperidin and naringin against 3-nitropropionic acid induced Huntington’s like symptoms in rats: Possible role of nitric oxide. Behav. Brain Res..

[56] Sachdeva A.K., Kuhad A., Chopra K. (2014). Naringin ameliorates memory deficits in experimental paradigm of Alzheimer’s disease by attenuating mitochondrial dysfunction. Pharmacol. Biochem. Behav..

[57] Cui Q.J., Wang L.Y., Wei Z.X., Qu W.S. (2014). Continual naringin treatment benefits the recovery of traumatic brain injury in rats through reducing oxidative and inflammatory alterations. Neurochem. Res..

[58] Chtourou Y., Slima A.B., Gdoura R., Fetoui H. (2015). Naringenin Mitigates Iron-Induced Anxiety-Like Behavioral Impairment, Mitochondrial Dysfunctions, Ectonucleotidases and Acetylcholinesterase Alteration Activities in Rat Hippocampus. Neurochem. Res..

[59] Frandsen J., Narayanasamy P. (2017). Flavonoid Enhances the Glyoxalase Pathway in Cerebellar Neurons to Retain Cellular Functions. Sci. Rep..

[60] Yen G.C., Duh P., Der Tsai H.L., Huang S.L. (2003). Pro-oxidative properties of flavonoids in human lymphocytes. Biosci. Biotechnol. Biochem..

[61] Matsuo M., Sasaki N., Saga K., Kaneko T. (2005). Cytotoxicity of flavonoids toward cultured normal human cells. Biol. Pharm. Bull..

[62] Halliwell B. (2009). The wanderings of a free radical. Free Radic. Biol. Med..

[63] Allaman I., Bélanger M., Magistretti P.J. (2015). Methylglyoxal, the dark side of glycolysis. Front. Neurosci..

[64] Haack F., Lemcke H., Ewald R., Rharass T., Uhrmacher A.M. (2015). Spatio-temporal Model of Endogenous ROS and Raft-Dependent WNT/Beta-Catenin Signaling Driving Cell Fate Commitment in Human Neural Progenitor Cells. PLoS Comput. Biol..

[65] Zheng X., Boyer L., Jin M., Mertens J., Kim Y., Ma L., Ma L., Hamm M., Gage F.H., Hunter T. (2016). Metabolic reprogramming during neuronal differentiation from aerobic glycolysis to neuronal oxidative phosphorylation. Elife.

[66] Park S.-Y., Kang M.-J., Han J.-S. (2017). Neuronal NOS Induces Neuronal Differentiation Through a PKCα-Dependent GSK3β Inactivation Pathway in Hippocampal Neural Progenitor Cells. Mol. Neurobiol..

[67] Park K.-Y., Na Y., Kim M.S. (2016). Role of Nox4 in Neuronal Differentiation of Mouse Subventricular Zone Neural Stem Cells. J. Life Sci..

[68] Olguín-Albuerne M., Morán J. (2015). ROS Produced by NOX2 Controls In Vitro Development of Cerebellar Granule Neurons Development. ASN Neuro.

[69] Yan Y., Wladyka C., Fujii J., Sockanathan S. (2015). Prdx4 is a compartment-specific H2O2 sensor that regulates neurogenesis by controlling surface expression of GDE2. Nat. Commun..

[70] Munnamalai V., Suter D.M. (2009). Reactive oxygen species regulate F-actin dynamics in neuronal growth cones and neurite outgrowth. J. Neurochem..

[71] Kim S.-U., Park Y.-H., Kim J.-M., Sun H.-N., Song I.-S., Huang S.M., Lee S.-H., Chae J.-I., Hong S., Sik Choi S. (2014). Dominant Role of Peroxiredoxin/JNK Axis in Stemness Regulation During Neurogenesis from Embryonic Stem Cells. Stem Cells.

[72] Wilson C., Muñoz-Palma E., González-Billault C. (2018). From birth to death: A role for reactive oxygen species in neuronal development. Semin. Cell Dev. Biol..

[73] Collins S.J., Tumpach C., Groveman B.R., Drew S.C., Haigh C.L. (2018). Prion protein cleavage fragments regulate adult neural stem cell quiescence through redox modulation of mitochondrial fission and SOD2 expression. Cell. Mol. Life Sci..

[74] Morinaka A., Yamada M., Itofusa R., Funato Y., Yoshimura Y., Nakamura F., Yoshimura T., Kaibuchi K., Goshima Y., Hoshino M. (2011). Thioredoxin mediates oxidation-dependent phosphorylation of CRMP2 and growth cone collapse. Sci. Signal..

[75] Chui A., Zhang Q., Dai Q., Shi S.-H. (2020). Oxidative stress regulates progenitor behavior and cortical neurogenesis. Development.

[76] Ji F., Shen T., Zou W., Jiao J. (2017). UCP2 Regulates Embryonic Neurogenesis via ROS-Mediated Yap Alternation in the Developing Neocortex. Stem Cells.

[77] Khacho M., Clark A., Svoboda D.S., MacLaurin J.G., Lagace D.C., Park D.S., Slack R.S. (2017). Mitochondrial dysfunction underlies cognitive defects as a result of neural stem cell depletion and impaired neurogenesis. Hum. Mol. Genet..

[78] Coyoy A., Olguín-Albuerne M., Martínez-Briseño P., Morán J. (2013). Role of reactive oxygen species and NADPH-oxidase in the development of rat cerebellum. Neurochem. Int..

[79] Schroeter H., Spencer J.P.E., Rice-Evans C., Williams R.J. (2001). Flavonoids protect neurons from oxidized low-density-lipoprotein-induced apoptosis involving c-Jun N-terminal kinase (JNK), c-Jun and caspase-3. Biochem. J..

[80] Kobuchi H., Roy S., Sen C.K., Nguyen H.G., Packer L. (1999). Quercetin inhibits inducible ICAM-1 expression in human endothelial cells through the JNK pathway. Am. J. Physiol. Physiol..

[81] Spencer J.P.E., Schroeter H., Kuhnle G., Srai S.K.S., Tyrrell R.M., Hahn U., Rice-Evans C. (2001). Epicatechin and its in vivo metabolite, 3′-O-methyl epicatechin, protect human fibroblasts from oxidative-stress-induced cell death involving caspase-3 activation. Biochem. J..

[82] Arola-Arnal A., Oms-Oliu G., Crescenti A., del Bas J.M., Ras M.R., Arola L., Caimari A. (2013). Distribution of grape seed flavanols and their metabolites in pregnant rats and their fetuses. Mol. Nutr. Food Res..

[83] Abd El Mohsen M., Marks J., Kuhnle G., Rice-Evans C., Moore K., Gibson G., Debnam E., Srai S.K. (2004). The differential tissue distribution of the citrus flavanone naringenin following gastric instillation. Free Radic. Res..

[84] Jochum F., Alteheld B., Meinardus P., Dahlinger N., Nomayo A., Stehle P. (2017). Mothers’ Consumption of Soy Drink But Not Black Tea Increases the Flavonoid Content of Term Breast Milk: A Pilot Randomized, Controlled Intervention Study. Ann. Nutr. Metab..

[85] Izquierdo V., Palomera-Ávalos V., López-Ruiz S., Canudas A.-M., Pallàs M., Griñán-Ferré C. (2019). Maternal Resveratrol Supplementation Prevents Cognitive Decline in Senescent Mice Offspring. Int. J. Mol. Sci..

[86] Bayer S.A., Altman J., Russo R.J., Zhang X. (1993). Timetables of neurogenesis in the human brain based on experimentally determined patterns in the rat. Neurotoxicology.

[87] Zielinsky P., Martignoni F.V., Vian I. (2014). Deleterious effects of maternal ingestion of cocoa upon fetal ductus arteriosus in late pregnancy. Front. Pharmacol..

[88] Torres-Cuevas I., Parra-Llorca A., Sánchez-Illana A., Nuñez-Ramiro A., Kuligowski J., Cháfer-Pericás C., Cernada M., Escobar J., Vento M. (2017). Oxygen and oxidative stress in the perinatal period. Redox Biol..

[89] Perrone S., Tataranno L.M., Stazzoni G., Ramenghi L., Buonocore G. (2015). Brain susceptibility to oxidative stress in the perinatal period. J. Matern. Neonatal Med..

[90] Inder T., Mocatta T., Darlow B., Spencer C., Volpe J.J., Winterbourn C. (2002). Elevated Free Radical Products in the Cerebrospinal Fluid of VLBW Infants with Cerebral White Matter Injury. Pediatr. Res..

[91] Khacho M., Clark A., Svoboda D.S., Azzi J., MacLaurin J.G., Meghaizel C., Sesaki H., Lagace D.C., Germain M., Harper M.-E. (2016). Mitochondrial Dynamics Impacts Stem Cell Identity and Fate Decisions by Regulating a Nuclear Transcriptional Program. Cell Stem Cell.

