# Supplementary material for: Naringin Supplementation during Pregnancy Induces Sex and Region-Specific Alterations in the Offspring’s Brain Redox Status
Source: Int J Environ Res Public Health. 2021 Apr 30;18(9):4805. doi: 10.3390/ijerph18094805 (PMC8124438; doi:10.3390/ijerph18094805)
Supplement: Supplementary file 1 [file ijerph-18-04805-s001.zip › ijerph-1138759-supplementary/Supplementary table S1.pdf]

Supplementary table S1: Statistical data from the biochemical analyses performed in the offspring's prefrontal cortex.

Prefrontal cortex

**Postnatal day 1**						
Parameter	***Supplementation effect***		***Sex effect***		***Interaction***	
	p value	***Statistical data***	***p value***	***Statistical data***	***p value***	***Statistical data***
2′,7′-dichlorofluorescein oxidation	0.770	F(1,34)=0.087	<0.001	F(1,34)=68.661	0.780	F(1,34)=0.079
Superoxide dismutase activity	0.392	F(1,32)=0.752	0.592	F(1,32)=0.292	0.956	F(1,32)=0.003
Glutathione peroxidase activity	0.024	F(1,31)=5.598	0.003	F(1,31)=10.710	0.330	F(1,31)=0.978
Catalase activity	0.610	F(1,34)=0.265	0.100	F(1,34)=2.854	0.157	F(1,34)=2.098

Glyoxalase activity	0.349	F(1,34)=0.901	0.048	F(1,34)=4.212	0.427	F(1,34)=0.648
Reduced glutathione content	0.669	F(1,23)=0.187	0.397	F(1,23)=0.746	0.972	F(1,23)=0.001
SOD/GPx ratio	0.886	F(1,26)=0.021	0.029	F(1,26)=5.378	0.563	F(1,26)=0.344
Postnatal day 7						
2′,7′-dichlorofluorescein oxidation	0.437	F(1,32)=0.620	<0.001	F(1,32)=15.350	0.190	F(1,32)=1.792
Superoxide dismutase activity	0.189	F(1,33)=1.795	0.981	F(1,33)=0.001	0.002	F(1,33)=11.050
Glutathione peroxidase activity	0.833	F(1,33)=0.045	0.004	F(1,33)=9.834	0.008	F(1,33)=8.050
Catalase activity	0.067	F(1,30)=3.607	0.038	F(1,30)=4.701	0.442	F(1,30)=0.606
Glyoxalase activity	0.787	F(1,35)=0.074	0.001	F(1,35)=13.239	0.026	F(1,35)=5.433
Reduced glutathione content	0.012	F(1,33)=7.095	0.110	F(1,33)=2.695	0.390	F(1,33)=0.758
SOD/GPx ratio	0.274	F(1,27)=1.249	0.038	F(1,27)=4.764	0.514	F(1,27)=0.436
Postnatal day 21						
2′,7′-dichlorofluorescein oxidation	0.359	F(1,26)=0.359	0.446	F(1,26)=0.599	0.887	F(1,26)=0.021

Superoxide dismutase activity	0.785	F(1,28)=0.076	0.011	F(1,28)=7.418	0.792	F(1,28)=0.071
Glutathione peroxidase activity	0.111	F(1,28)=2.712	0.009	F(1,28)=7.934	0.565	F(1,28)=0.340
Catalase activity	0.449	F(1,25)=0.591	0.188	F(1,25)=1.828	0.084	F(1,25)=3.243
Glyoxalase activity	0.541	F(1,28)=0.383	0.194	F(1,28)=1.772	0.548	F(1,28)=0.370
Reduced glutathione content	0.481	F(1,25)=0.511	0.362	F(1,25)=0.862	0.717	F(1,25)=0.135
SOD/GPx ratio	0.022	F(1,23)=6.063	0.944	F(1,23)=0.005	0.790	F(1,23)=0.072
