# Supplementary material for: Naringin Supplementation during Pregnancy Induces Sex and Region-Specific Alterations in the Offspring’s Brain Redox Status
Source: Int J Environ Res Public Health. 2021 Apr 30;18(9):4805. doi: 10.3390/ijerph18094805 (PMC8124438; doi:10.3390/ijerph18094805)
Supplement: Supplementary file 1 [file ijerph-18-04805-s001.zip › ijerph-1138759-supplementary/Supplementary table S2.pdf]

Supplementary table S2: Statistical data from the biochemical analyses performed in the offspring's hippocampus.

Hippocampus

**Postnatal day 1**						
Parameter	***Supplementation effect***		***Sex effect***		***Interaction***	
	p value	***Statistical data***	***p value***	***Statistical data***	***p value***	***Statistical data***
2′,7′-dichlorofluorescein oxidation	0.863	F(1,37)=0.030	0.006	F(1,37)=8.636	0.860	F(1,37)=0.031
Superoxide dismutase activity	0.795	F(1,39)=0.068	0.014	F(1,39)=6.680	0.260	F(1,39)=1.307
Glutathione peroxidase activity	0.001	F(1,42)=12.539	<0.001	F(1,42)=16.927	0.934	F(1,42)=0.007
Catalase activity	0.078	F(1,33)=3.307	0.092	F(1,33)=3.021	0.931	F(1,33)=0.008

Glyoxalase activity	0.152	F(1,36)=2.143	0.159	F(1,36)=2.066	0.071	F(1,36)=3.470
Reduced glutathione content	0.003	F(1,29)=10.193	0.002	F(1,29)=11.897	0.981	F(1,29)=0.001
SOD/GPx ratio	0.631	F(1,31)=0.235	0.324	F(1,31)=1.004	0.152	F(1,31)=2.155
Postnatal day 7						
2′,7′-dichlorofluorescein oxidation	0.184	F(1,36)=1.839	<0.001	F(1,36)=28.105	0.314	F(1,36)=1.044
Superoxide dismutase activity	0.314	F(1,35)=1.049	0.101	F(1,35)=2.836	0.829	F(1,35)=0.047
Glutathione peroxidase activity	0.026	F(1,34)=5.422	<0.001	F(1,34)=36.052	0.680	F(1,34)=0.173
Catalase activity	0.052	F(1,34)=4.063	0.535	F(1,34)=0.392	0.864	F(1,34)=0.030
Glyoxalase activity	<0.001	F(1,35)=16.209	0.548	F(1,35)=0.392	0.809	F(1,35)=0.059
Reduced glutathione content	0.002	F(1,35)=11.822	0.419	F(1,35)=0.670	0.854	F(1,35)=0.034
SOD/GPx ratio	0.013	F(1,33)=6.884	0.005	F(1,33)=9.251	0.416	F(1,33)=0.677
Postnatal day 21						
2′,7′-dichlorofluorescein oxidation	0.905	F(1,29)=0.014	0.045	F(1,29)=4.393	0.743	F(1,29)=0.109

Superoxide dismutase activity	0.315	F(1,30)=1.045	0.262	F(1,30)=1.309	0.556	F(1,30)=0.354
Glutathione peroxidase activity	0.481	F(1,30)=0.508	0.480	F(1,30)=0.511	0.703	F(1,30)=0.148
Catalase activity	0.214	F(1,30)=1.611	0.037	F(1,30)=4.755	0.353	F(1,30)=0.891
Glyoxalase activity	0.039	F(1,30)=4.671	0.990	F(1,30)=0.000	0.511	F(1,30)=0.442
Reduced glutathione content	0.524	F(1,30)=0.416	0.006	F(1,30)=8.621	0.279	F(1,30)=1.215
SOD/GPx ratio	0.363	F(1,29)=0.854	0.149	F(1,29)=2.199	0.714	F(1,29)=0.137
