# Supplementary material for: Naringin Supplementation during Pregnancy Induces Sex and Region-Specific Alterations in the Offspring’s Brain Redox Status
Source: Int J Environ Res Public Health. 2021 Apr 30;18(9):4805. doi: 10.3390/ijerph18094805 (PMC8124438; doi:10.3390/ijerph18094805)
Supplement: Supplementary file 1 [file ijerph-18-04805-s001.zip › ijerph-1138759-supplementary/Supplementary table S3.pdf]

Supplementary table S3: Statistical data from the biochemical analyses performed in the offspring's striatum.

Striatum

**Postnatal day 1**						
Parameter	***Supplementation effect***		***Sex effect***		***Interaction***	
	p value	***Statistical data***	***p value***	***Statistical data***	***p value***	***Statistical data***
2′,7′-dichlorofluorescein oxidation	0.007	F(1,36)=8.249	<0.001	F(1,36)=41.348	0.058	F(1,36)=3.846
Superoxide dismutase activity	0.340	F(1,36)=0.340	0.668	F(1,36)=0.187	0.839	F(1,36)=0.042
Glutathione peroxidase activity	0.092	F(1,36)=2.989	0.187	F(1,36)=1.813	0.704	F(1,36)=0.146
Catalase activity	0.221	F(1,36)=1.554	0.444	F(1,36)=0.600	0.775	F(1,36)=0.083

Glyoxalase activity	0.988	F(1,36)=0.000	0.885	F(1,36)=0.021	0.798	F(1,36)=0.066
Reduced glutathione content	0.345	F(1,31)=0.918	0.745	F(1,31)=0.107	0.724	F(1,31)=0.127
SOD/GPx ratio	0.921	F(1,36)=0.010	0.272	F(1,36)=1.244	0.938	F(1,36)=0.006
Postnatal day 7						
2′,7′-dichlorofluorescein oxidation	0.214	F(1,32)=1.609	0.003	F(1,32)=10.552	p=0.008	F(1,32)=7.865
Superoxide dismutase activity	0.326	F(1,35)=0.991	0.002	F(1,35)=10.962	0.079	F(1,35)=3.275
Glutathione peroxidase activity	0.003	F(1,35)=10.199	0.347	F(1,35)=0.347	0.595	F(1,35)=0.288
Catalase activity	0.272	F(1,36)=1.242	0.937	F(1,36)=0.006	0.049	F(1,36)=4.146
Glyoxalase activity	0.033	F(1,35)=16.435	<0.001	F(1,35)=16.435	0.020	F(1,35)=5.940
Reduced glutathione content	0.062	F(1,29)=14.844	0.798	F(1,29)=0.067	0.839	F(1,29)=0.042
SOD/GPx ratio	0.001	F(1,33)=14.746	0.057	F(1,33)=3.898	0.115	F(1,33)=2.626
Postnatal day 21						
2′,7′-dichlorofluorescein oxidation	0.623	F(1,34)=0.246	0.113	F(1,34)=2.652	0.093	F(1,34)=0.006

Superoxide dismutase activity	0.981	F(1,28)=0.001	0.742	F(1,28)=0.111	0.235	F(1,28)=1.473
Glutathione peroxidase activity	0.112	F(1,31)=2.669	0.436	F(1,31)=0.624	0.944	F(1,31)=0.005
Catalase activity	0.344	F(1,31)=0.923	0.321	F(1,31)=1.015	0.093	F(1,31)=3.006
Glyoxalase activity	0.831	F(1,34)=0.046	0.907	F(1,34)=0.014	0.591	F(1,34)=0.295
Reduced glutathione content	0.042	F(1,34)=4.448	0.222	F(1,34)=1.550	0.496	F(1,34)=0.473
SOD/GPx ratio	0.255	F(1,23)=1.361	0.956	F(1,23)=0.003	0.175	F(1,23)=1.958
