# Supplementary material for: Naringin Supplementation during Pregnancy Induces Sex and Region-Specific Alterations in the Offspring’s Brain Redox Status
Source: Int J Environ Res Public Health. 2021 Apr 30;18(9):4805. doi: 10.3390/ijerph18094805 (PMC8124438; doi:10.3390/ijerph18094805)
Supplement: Supplementary file 1 [file ijerph-18-04805-s001.zip › ijerph-1138759-supplementary/Supplementary table S4.pdf]

**Supplementary table S4:** Statistical data from the biochemical analyses performed in the offspring's cerebellum.

**Cerebellum**

| **Postnatal day 1** | | | | | | |
|---|---|---|---|---|---|---|
| ***Parameter*** | ***Supplementation effect*** | | ***Sex effect*** | | ***Interaction*** | |
| | ***p value*** | ***Statistical data*** | ***p value*** | ***Statistical data*** | ***p value*** | ***Statistical data*** |
| 2′,7′-dichlorofluorescein oxidation | 0.332 | F(1,40)=0.963 | 0.641 | F(1,40)=0.221 | 0.967 | F(1,40)=0.002 |
| Superoxide dismutase activity | 0.282 | F(1,36)=1.195 | <0.001 | F(1,36)=15.604 | 0.799 | F(1,36)=0.066 |
| Glutathione peroxidase activity | 0.152 | F(1,43)=2.127 | 0.004 | F(1,43)=9.085 | 0.792 | F(1,43)=0.070 |
| Catalase activity | 0.838 | F(1,39)=0.042 | <0.001 | F(1,39)=18.112 | 0.631 | F(1,39)=0.234 |

| | | | | | | |
|---|---|---|---|---|---|---|
| Glyoxalase activity | 0.630 | F(1,36)=0.236 | 0.521 | F(1,36)=0.421 | 0.414 | F(1,36)=0.683 |
| Reduced glutathione content | 0.263 | F(1,34)=1.297 | 0.003 | F(1,34)=10.543 | 0.263 | F(1,34)=1.297 |
| SOD/GPx ratio | 0.545 | F(1,33)=0.373 | <0.001 | F(1,33)=30.743 | 0.338 | F(1,33)=0.946 |
| ***Postnatal day 7*** | | | | | | |
| 2′,7′-dichlorofluorescein oxidation | 0.003 | F(1,36)=10.109 | <0.001 | F(1,36)=196.026 | 0.021 | F(1,36)=5.848 |
| Superoxide dismutase activity | 0.001 | F(1,36)=13.953 | <0.001 | F(1,36)=24.983 | 0.470 | F(1,36)=0.533 |
| Glutathione peroxidase activity | 0.012 | F(1,36)=6.941 | <0.001 | F(1,36)=30.964 | 0.485 | F(1,36)=33.507 |
| Catalase activity | 0.420 | F(1,34)=0.667 | 0.243 | F(1,34)=1.409 | 0.476 | F(1,34)=0.518 |
| Glyoxalase activity | 0.015 | F(1,36)=6.595 | 0.776 | F(1,36)=0.082 | 0.494 | F(1,36)=0.477 |
| Reduced glutathione content | 0.061 | F(1,36)=3.751 | 0.002 | F(1,36)=10.597 | 0.183 | F(1,36)=1.847 |
| SOD/GPx ratio | 0.109 | F(1,33)=2.717 | <0.001 | F(1,33)=119.267 | 0.335 | F(1,33)=0.958 |
| ***Postnatal day 21*** | | | | | | |
| 2′,7′-dichlorofluorescein oxidation | 0.697 | F(1,28)=0.155 | 0.022 | F(1,28)=5.892 | 0.590 | F(1,28)=0.297 |

| | | | | | | |
|---|---|---|---|---|---|---|
| Superoxide dismutase activity | 0.090 | F(1,28)=3.092 | 0.653 | F(1,28)=0.206 | 0.035 | F(1,28)=4.921 |
| Glutathione peroxidase activity | 0.637 | F(1,28)=0.228 | 0.006 | F(1,28)=8.785 | 0.083 | F(1,28)=3.227 |
| Catalase activity | 0.063 | F(1,28)=3.763 | 0.763 | F(1,28)=0.092 | 0.324 | F(1,28)=1.010 |
| Glyoxalase activity | 0.016 | F(1,28)=6.559 | 0.004 | F(1,28)=9.643 | 0.031 | F(1,28)=5.163 |
| Reduced glutathione content | 0.783 | F(1,27)=0.077 | 0.004 | F(1,27)=10.187 | 0.298 | F(1,27)=1.127 |
| SOD/GPx ratio | 0.187 | F(1,27)=1.837 | 0.006 | F(1,27)=8.714 | 0.889 | F(1,27)=0.020 |
